# Factors Associated with Binge Eating Behavior among Malaysian Adolescents

**DOI:** 10.3390/nu10010066

**Published:** 2018-01-10

**Authors:** Wan Ying Gan, Normasliana Mohamad, Leh Shii Law

**Affiliations:** Department of Nutrition and Dietetics, Faculty of Medicine and Health Sciences, Universiti Putra Malaysia, Serdang 43400, Malaysia; maslianamohamad@gmail.com (N.M.); lehshii@gmail.com (L.S.L.)

**Keywords:** binge eating behavior, adolescent, depression, obesity, body dissatisfaction

## Abstract

Although there are numerous studies on binge eating behavior in the Western countries, studies on this behavior in Malaysia are still limited. Therefore, this cross-sectional study aimed to determine the risk factors associated with binge eating behavior among adolescents in Malaysia. The study included 356 adolescents (42.7% males and 57.3% females), aged 13 to 16 years. They completed a self-administered questionnaire on demographic and socioeconomic backgrounds, frequency of family meals, family meal environments, family cohesion, perception of body size, self-esteem, depressive symptoms, perfectionistic self-presentation, and binge eating behavior. Furthermore, their weight, height, and waist circumference were measured. It was found that 14.0% of the participants engaged in binge eating behavior (15.2% in females and 12.5% in males). Additionally, it was identified that high levels of depressive symptoms, high levels of body dissatisfaction, poor family cohesion, and low self-esteem were significantly contributed to binge eating behavior after controlling for sex (adjusted *R*^2^ = 0.165, *F* = 15.056, *p* < 0.001). The findings may suggest that improving the relationships between family members, along with eliminating adolescents’ negative emotions could help in the prevention of binge eating behavior among adolescents. The identified modifiable risk factors should be incorporated into binge eating preventive programs to increase the effectiveness of the programs.

## 1. Introduction

Binge eating behavior has been described as the consumption of large amounts of food that most people would not have consumed in a specific duration of time. Furthermore, the affected individuals have no control over their overeating behaviors [1]. Noticeable examples of binge eating behavior are eating rapidly, eating until one is uncomfortably full, eating while one is not hungry, eating alone due to embarrassment, and feeling disgusted or guilty with oneself. The difference between binge eating behavior and binge eating disorder is that the latter is recognized as a psychological disorder. On the other hand, binge eating behavior is known as a behavioral symptom of a binge eating disorder. In summary, binge eating behavior is antecedent to binge eating disorder [2]. It was found that groups that were vulnerable to the development of binge eating behavior included both males and females from different age groups, including those in their childhood [3,4], during adolescence [5,6], and adulthood [7,8]. Therefore, the current study was mainly focused on binge eating behavior, a preliminary stage of binge eating disorder.

Adults with binge eating disorder had engaged with binge eating behavior since their adolescence [9]. In order to achieve optimal growth and development during adolescence, the nutritional requirements of adolescents are the highest across the life span. Thus, healthy eating behaviors among adolescents are important to meet their nutritional needs. Binge eating behavior was found to be prevalent among adolescents and has been a public health concern with serious physical and mental health consequences. Previous studies found that binge eating was associated with obesity and other psychiatric disorders, such as depression and anxiety disorders [10], substance abuse [10,11], and compulsive behaviors, such as gambling [11] and binge drinking [12]. If binge eating behavior among adolescents is well understood, it might help to identify those at risk among them, so that suitable preventive treatments could be administered to individuals in the early stages of binge eating development. The prevention could be administered before the affected individual suffers from the physical and mental negative effects of binge eating behavior. It was found that people affected by binge eating behavior had a higher chance of recovery when early detection and treatment occurred [13]. Nevertheless, stigma related to binge eating is an obstacle that prevents affected individuals from seeking help. The stigma occurred because people with a risk of developing eating behavior problems normally feel shame, ambivalence, and denial [13].

Binge eating behavior has become a nutritional concern. Data from a 10-year longitudinal study, Project EAT (Eating among Teens and Young Adults), showed that the prevalence of binge eating in females increased from 9.9% during middle adolescence to 14.1% in middle young adulthood, whereas the prevalence increased from 3.0% in middle adolescence to 5.9% in middle young adulthood among males [14]. Similarly, a seven-year Growing Up Today Study (GUTS) revealed that binge eating increased with age during adolescence from 2.4% to 5.7% between 1999 to 2003 [15]. In Malaysia, there is limited study on binge eating behavior. The prevalence of binge eating behavior was reported to be 4.1% among adolescents in Selangor [16]. Another local study that was conducted on secondary school girls revealed a much higher prevalence rate at 35.4% [17]. The significant difference in the prevalence rates of binge eating behavior found among adolescents in Malaysia was abnormal and contradictory. The contradiction between studies warranted the need for future studies.

There is no doubt that unhealthy eating behaviors are fast spreading to Asia, and Malaysia is no exception due to increasing globalization and exposure to Western culture. Understanding binge eating behavior is a vital step in the formulation of strategies to reduce the incidence of unhealthy eating behaviors among adolescents. Previous studies have found that there were a number of factors that are associated with binge eating behavior among adolescents, which included: demographic factors (such as sex and ethnicity [10]), behavioral factors (emotional and social eating, skipping meals, snacking, eating sweets [18], unbalanced diets [19], and secretive eating [20]), nutritional status (overweight/obesity [21]), psychological factors (depression, body dissatisfaction [19,21,22], perceived stress and anxiety [23], low self-esteem [22], a lack of interoceptive awareness, and internalization of the sociocultural body ideals [24]), as well as environmental factors (weight teasing from peers and family [25,26]).

Binge eating behavior requires attention, as the behavior is highly correlated to several negative health effects not only in adolescents, but in adults as well. The health effects included a distorted health (a lower quality of life [27]), the development of diseases, such as gastric dilatation [28], idiopathic intracranial hypertension, gastric perforation [29], and irritable bowel syndrome [30]), an unhealthy body composition [31] (a higher body mass index, higher waist circumference, higher fat mass, and lower lean mass), and an unfavorable metabolic and inflammatory profile [31] (lower high-density lipoprotein cholesterol, higher glycated hemoglobin, higher uric acid, higher erythrocyte sedimentation rate, higher sensitive C-reactive protein, higher white blood cell count, higher fasting insulin, and higher insulin resistance).

The relationships between family factors, psychological factors and body weight status with binge eating behavior among Malaysian adolescents remain unclear. This unhealthy eating behavior may be different from Western countries due to social and cultural differences. Therefore, this study investigated factors associated with binge eating behavior among adolescents in Malaysia, to test the hypothesis that less frequent family meals, poor family meal environments, poor family cohesion, body image dissatisfaction, depressive symptoms, low self-esteem, and low level of perfectionistic self-presentation increased the risk of binge eating behavior among adolescents.

## 2. Materials and Methods

### 2.1. Participants

The participants were recruited from two (out of fifteen) randomly selected secondary public schools in Kajang, a district in Selangor state, Malaysia. Twelve (out of thirty) Grade 8 to 10 classes from two schools were randomly selected. All of the students aged 13 to 16 years old in the selected classes were invited to participate in this study (*n* = 389). Only students who had the informed consent of their parents were involved in this study. Furthermore, those students with physical and/or mental health conditions or the presence of a chronic disease were excluded from this study because researchers were not able to measure their height and weight and they might have different levels of psychological well-being.

### 2.2. Ethical Clearance and Permission

Ethical approval was granted from the Ethics Committee for Research Involving Human Subjects of Universiti Putra Malaysia (Reference No.: FPSK/November(13)13). In addition, approvals to conduct research activities in schools were obtained from the Malaysia Ministry of Education, Selangor Department of Education, and the selected schools. Written informed consents were obtained from the participants and their parents prior to data collection.

### 2.3. Measures

A Malay language self-administered questionnaire was used to collect data and was completed by the participants in their classrooms with the assistance of the researchers. The validated Malay versions of the Binge Eating Scale [32], Family Environment Scale [33], Rosenberg Self-esteem Scale [34], and Center for Epidemiological Studies-Depressed Mood Scale [35] were used in this study. All of the other scales used in this study were translated into Malay language by two experts who are fluent in both the English and Malay languages and back-translated into English by another bi-lingual expert.

#### 2.3.1. Demographic and Socioeconomic Characteristics

Information regarding demographic and socioeconomic characteristics included age, sex, ethnicity, household size, education of parents, occupation of parents, and household income.

#### 2.3.2. Binge Eating Behavior

The 16-item Binge Eating Scale [36] was used to assess the presence of binge eating behavior (8-item), as well as feelings and thoughts that are associated with such behavior (8-item). The total score for binge eating behavior ranged between 0 and 46. Participants who scored ≤17 were considered to be non-binge eaters. Moreover, a score between 18 and 26 was categorized as moderate binge eating behavior and a score ≥27 was categorized as severe binge eating behavior. Participants who fell in the moderate and severe binge eating categories were considered to have binge eating problems. The Binge Eating Scale has been found to have the capability to distinguish individuals with none, moderate, or severe binge eating problems well [36]. The scale has also been shown to have good reliability and validity [36]. The scale used within this study showed good internal consistency (Cronbach’s α = 0.79).

#### 2.3.3. Frequency of Family Meals

The factor: frequency of family meals at home, was assessed with the question ‘*During the past seven days, how many times did all, or most, of your family living in your house eat a meal together?*’ [37]. A 6-point Likert scale was used which provided participants with the options to answer: ‘never’, ‘1 to 2 times’, ‘3 to 4 times’, ‘5 to 6 times’, ‘7 times’, and ‘>7 times’. Nevertheless, the last three categories were merged and labelled ‘5 or more times’ to simplify the analysis of data.

#### 2.3.4. Family Meal Environment

The participants were required to express their degree of agreement with 14 items that accurately described their family mealtimes. The items included priority of family meals (five items), atmosphere at family meals (four items), and structure/rules at family meals (five items) [37]. The participants were provided with a 4-point Likert scale (‘strongly agree’, ‘agree’, ‘disagree’, and ‘strongly disagree’) as options for answers. For positive statements, ‘strongly agree’, ‘agree’, ‘disagree’, and ‘strongly disagree’ were given a score of 4, 3, 2, and 1, respectively. On the other hand, the scoring for negative statements was reversed. This scale demonstrated good internal consistency (Cronbach’s α = 0.73) in this study.

#### 2.3.5. Family Cohesion

A family cohesion subscale from the Family Environment Scale was used to assess the interpersonal atmosphere within a family with respect to relationships among family members [38]. The family cohesion scale consisted of nine items. Each item was prepared with the responses: ‘mostly true’ and ‘mostly false’. If a participant selected ‘mostly true’, a score of 1 was assigned to the item. The total score for the scale ranged between 0 and 9. A higher score indicated a more cohesive family environment. The scale in this study demonstrated good internal consistency (Cronbach’s α = 0.76).

#### 2.3.6. Perception of Body Size

The Contour Drawing Rating Scale [39] was used to assess perception of body size and overall body size satisfaction levels. The scale was a pictorial instrument containing a continuum of nine developed male and female body figures. The body figures were presented in ascending order from severely underweight (score 1) to extremely overweight (score 9). The participants were requested to choose the drawing that most accurately depicted their current body size and their most desired body size. The discrepancy between current and desired size is an index of the participants’ overall body size satisfaction level. A positive discrepancy score indicated that participants desired to have a larger body size. A discrepancy score of ‘zero’ indicated participants desired to maintain their current body size. Finally, a negative discrepancy score indicated participants desired to have a smaller body size. This scale showed good test-retest reliability and construct validity [39].

#### 2.3.7. Self-Esteem

A 10-item Rosenberg Self-Esteem Scale [40] with a 4-point Likert scale (‘strongly agree’, ‘agree’, ‘disagree’, and ‘strongly disagree’) was used to assess the self-esteem of participants. The option ‘strongly agree’ was given a score of 3, ‘agree’ was given a score of 2, ‘disagree’ was given a score of 1, and ‘strongly disagree’ was given a score of 0. The scoring for items 2, 5, 6, 8 and 9 was reversed. The total score that could be attained in the scale ranged between 0 and 30. A higher score indicated a higher level of self-esteem. The internal consistency in this study for this scale was good (Cronbach’s α = 0.73).

#### 2.3.8. Depressive Symptoms

The 20-item Center for Epidemiological Studies-Depressed Mood Scale (CES-D) [41] was used to measure symptoms, such as depressed moods, feelings of guilt and worthlessness, feelings of helplessness and hopelessness, psychomotor retardation, loss of appetite, and sleep disturbance. The responses in the scale were based on the frequency of occurrence of each symptom. The responses that could be selected were: ‘rarely or none of the time’ (less than 1 day), ‘some or a little of the time’ (1 to 2 days), ‘occasionally of a moderate amount of time’ (3 to 4 days), and ‘most or all of the time’ (5 to 7 days). The score for each response on a negative item ranged between 0 and 3. Nonetheless, the scoring was reversed for positive items (items 4, 8, 12, and 16). The total score ranged between 0 and 60. A higher CES-D score indicated greater depressive symptoms, which increased the risk of depression. An indication of significant depressive symptoms was a score of 16 or more [41]. The scale in the present study demonstrated excellent internal consistency (Cronbach’s α = 0.88).

#### 2.3.9. Perfectionistic Self-Presentation

The Perfectionistic Self-Presentation Scale (PSPS) [42] was used to assess the participants’ need to appear perfect to others and hide defects or difficulties. The scale consists of 27 items and includes a seven-point Likert scale (strongly agree to strongly disagree). The participants could score between 1 and 7 depending on their agreement with each statement. The response strongly disagree scored 1, while strongly agree scored 7. Five items (1, 11, 16, 18, and 22) were reversely scored. The total score of PSPS ranged between 0 and 189. A higher score indicated a higher level of perfectionistic self-presentation of a participant. The scale in this study demonstrated excellent internal consistency (Cronbach’s α = 0.82).

#### 2.3.10. Anthropometric Measurements

Body weight, height and waist circumference (WC) of the participants were measured using three different tools, which were a TANITA Digital Weighing Scale HD-314 (TANITA Corporation, Arlington Heights, IL, USA), a SECA Body Tape Measure SE 206 (SECA, Hamburg, Germany), and a SECA 201 measuring tape (SECA, Hamburg, Germany), respectively. Each measurement was measured twice to get an average value. The *z*-score for BMI-for-age was computed according to the WHO Growth Reference 2007 [43] using the WHO AnthroPlus software version 1.0.4 (WHO, Geneva, Switzerland). According to the WC percentile charts for Malaysian children, a WC > 90th percentile was defined as abdominal obesity [44].

### 2.4. Data Analyses

Data analysis was conducted using the IBM SPSS Statistics 22.0 (IBM Corp., Armonk, NY, USA). Descriptive data for continuous variables were presented via means (M) and standard deviations (SD). On the other hand, details on categorical data were shown via counts (*n*) and percentages (%). An independent-samples *t*-test was used to determine the mean difference of binge eating behavior and body weight status between male and female participants. A simple linear regression was used to test the associations between continuous variables (i.e., age, household size, monthly household income, priority of family meals, atmosphere at family meals, structure at family meals, family cohesion, perception of body size, depressive symptoms, self-esteem, perfectionistic self-presentation, BMI-for-age, and waist circumference), and binge eating behavior. Additionally, a multiple linear regression analysis (stepwise method) was used [45] to determine the factors that contributed to binge eating behavior. Only variables that resulted in a *p*-value less than 0.25 during the simple linear regression were selected for the multiple linear analysis. The level of statistical significance was set at *p* < 0.05.

## 3. Results

A total of 356 out of 389 adolescents participated in this study, yielding a response rate of 92%. Characteristics of the participants are shown in Table 1. A total of 356 participants (42.7% males and 57.3% females) with a mean age of 14.3 years (SD = 1.0 years) participated in this study. Two thirds of the participants were Malay (68.0%), followed by Indian (14.6%), Chinese (13.8%), and other ethnic groups (3.6%). A majority of the fathers attained a tertiary education (50.6%), while half of the mothers attained a secondary education (50.1%). The mean monthly household income was RM 5566.30 (SD = 5470.50).

As shown in Table 2, the prevalence of overweight and obese participants in this study was 16.9% and 10.9%, respectively. About one fifth (19.7%) of the boys were overweight and 10.5% were obese. It was found that the prevalence of overweight and obese girls was lower than boys. Overweight and obese girls were reported to be 14.7% and 11.3%, respectively. In terms of abdominal obesity, 14.6% of the participants were at risk of abdominal obesity, with a higher prevalence found in girls (15.2%) when compared to boys (13.8%). The mean binge eating score was 10.32 (SD = 6.29). Girls (M = 10.93, SD = 6.06) were found to have a significantly higher mean BES score (*t* = 2.116, *p* = 0.035) than boys (M = 9.51, SD = 6.53). Overall, the prevalence rate of moderate to severe binge eating behavior was 14%. Moreover, more girls (15.2%) than boys (12.5%) were engaged in binge eating behavior.

As shown in Table 3, the multiple linear regression analysis (stepwise method) illustrated that depressive symptoms (*β* = 0.194, *p* < 0.001), family cohesion (*β* = −0.206, *p* < 0.001), perceptions of body size (*β* = 0.157, *p* = 0.002), and self-esteem (*β* = −0.154, *p* = 0.003) were found to significantly contribute to binge eating behavior among adolescents, after controlling for sex, as results showed that binge eating behavior was significantly different between males and females in this study. The prediction model was statistically significant (*F* = 15.056, *p* < 0.001), and the four significant factors mentioned above accounted for 16.5% of the variance in binge eating behavior.

## 4. Discussion

The findings in this study provided evidence regarding the existence of binge eating behavior among adolescents in Malaysia. The prevalence rate of this study was lower than the prevalence rate found for Brazilian adolescents [14]. Conversely, the prevalence rate of this study was found to be higher than the prevalence rate of American adolescents [15]. Nonetheless, it is important to note that the stark contrast in data from different countries was due to the use of different criteria to define binge eating behavior. Furthermore, there were cultural differences that could very well explain the disparity between the data. Asians were less likely to endorse the symptoms of binge eating when compared to their Western counterparts due to cultural differences in symptom experience or reporting, where this could lead to underreporting of binge eating behavior in Asians [46]. The current study found that perceived sociocultural pressures of the participants did not determine binge eating behavior, indicating that, when compared to their counterparts in foreign countries, the participants in this study may have not been exposed to as many negative comments from their external environment, such as peers or media, regarding their weight or body size. School students in Malaysia have their code of attire in which their clothes have to cover their bodies and must be loose and thick so as not to expose their body shape.

It was found in this study that adolescent girls were more likely to engage in binge eating behaviors compared to their male counterparts. An American national representative study reported similar findings and found that adolescent girls were 18% more likely to encounter a loss of control (i.e., eating while not hungry) and 3.29 times more likely to be distressed (i.e., they were afraid of gaining weight during binge eating) when compared to male adolescents [10]. An explanation for this finding could be that males were less likely to report their binge eating behavior because it was less socially acceptable and unexpected for males to express their feelings. In addition, binge eating behavior has been frequently viewed as an eating problem that is related to females. Thus, males might have felt reluctant to admit that a binge eating episode was a problem [10]. In contrast, another study from the United States showed that binge eating behavior among obese adolescents did not differ by sex [47].

Family cohesion was associated with binge eating behavior among adolescents in this study. The finding was consistent with findings on overweight American adolescents who reported to engage in overeating and had a lower score for family cohesion [48]. The 7-year Growing Up Today Study (GUTS) also revealed the importance of family cohesion in influencing the occurrence of problematic eating behaviors. Indeed, it was found that a higher family cohesion and higher quality of parental relationships were significantly associated with a lower likelihood of developing problematic eating behaviors among male and female adolescents [49]. The reason for this is, high levels of family cohesion may help adolescents to adopt a healthful behavior and have a good psychological well-being. As found in another study, individuals living under less stressful environments were protected from engaging in unhealthy eating behaviors [50].

The current study found that factors such as negative body image perceptions, high depressive symptoms, and a low self-esteem contributed positively to binge eating behavior among adolescents. The findings were consistent with previous literature that suggested psychological factors, such as body image perceptions [21], depression [21,27], and self-esteem [24,51] should be paid attention to when dealing with binge eating behavior among adolescents. One possible explanation for the relationship between the factors and binge eating behavior was that body image dissatisfaction may trigger depression and low self-esteem. As a result, binge eating behavior was used as an attempt to regulate negative emotions [52,53]. Nevertheless, the interaction between body image perceptions and binge eating behavior was complex and did not appear to be a direct and linear relationship. Therefore, further studies are recommended.

The current study has several limitations. Firstly, a causal relationship between predictors (demographic and socioeconomic factors, family factors, psychological factors and body weight status) and binge eating behavior among adolescents could not be established due to the use of a cross-sectional study design. Future cohort studies should be carried out to provide more evidence regarding the relationships between the factors with binge eating behavior among adolescents. For example, a longitudinal study done by Pearson, Zapolski and Smith [54] found that negative affect predicted depression, which in turn predicted binge eating behavior in children with no sex difference was reported. However, due to limited studies on binge eating behavior among Malaysian adolescents, the cross-sectional study design was used. This study could provide preliminary findings about binge eating behavior among adolescents in order for future cohort studies to be conducted. Secondly, the information gathered through the self-reported questionnaire were at risk of being under or over reported due to self-presentational bias. Participants may have wanted to present themselves in a positive light, and thus they may have under or over reported this information. Additionally, limitations of the questionnaires for family variable need to be taken into account. The use of not validated questionnaires for family variable might affect the conclusion drawn from the data. It is recommended that a valid and reliable questionnaire for family variable to be used in future studies. Furthermore, this study is limited by the small sample size and involving only adolescents in the state of Selangor. Thus, the results cannot be generalized to all Malaysian adolescents. It is recommended that further localized studies or even national representative studies could be conducted in the future. Further studies in this area will ensure that binge eating behavior of adolescents in Malaysia could be well monitored.

## 5. Conclusions

The present study supported existing literature that found binge eating behavior was a problem among adolescents. Furthermore, this study found that adolescents who were dissatisfied with their body size, had problems interacting with family members, had high depressive symptoms, and low self-esteem, were at a higher risk of developing binge eating behavior. The study suggested a few actions that might prevent adolescents from engaging in binge eating behavior, which included an increase in interactions between family members, the promotion of positive body image and higher self-esteem, and the prevention of depression. Future researchers and public health practitioners who are involved in planning intervention programs on binge eating behavior among adolescents could incorporate the above mentioned actions in order to increase the effectiveness of intervention programs. Psychologists, counsellors, nutritionists, and school teachers should also work together to improve eating behaviors, nutritional status, and the mental health of adolescents.

## Figures and Tables

**Table 1:** Demographic and socioeconomic characteristics, family factors, and psychological factors of the participants (*n* = 356).

Characteristics	Mean ± SD	*n* (%)
Sex		
Males		152 (42.7)
Females		204 (57.3)
Age (years)	14.30 ± 1.04	
13–14		144 (40.5)
15–16		212 (59.6)
Ethnicity		
Malay		242 (68.0)
Chinese		49 (13.8)
Indian		52 (14.6)
Others		13 (3.6)
Household size (members)		
≤5		294 (57.3)
>5		152 (42.7)
Father’s education level		
Primary		11 (3.2)
Secondary		159 (45.9)
Tertiary		175 (50.6)
No schooling		1 (0.3)
Mother’s education level		
Primary		17 (4.8)
Secondary		177 (50.1)
Tertiary		157 (44.5)
No schooling		2 (0.6)
Monthly household income (RM)	5566.30 ± 5470.50	
<1000		19 (5.3)
1000–2999		91 (25.6)
3000–4999		84 (23.6)
5000–6999		67 (18.8)
7000–8999		37 (10.4)
≥9000		58 (16.3)
Priority of family meal	9.34 ± 3.16	
Atmosphere at family meals	8.33 ± 2.43	
Structure at family meals	7.97 ± 2.09	
Family cohesion	5.79 ± 1.32	
Perception of body size	0.29 ± 1.46	
Depressive symptoms	20.97 ± 11.22	
Self-esteem	28.16 ± 4.43	
Perfectionistic self-presentation	90.17 ± 17.16	

RM = Ringgit Malaysia; SD = standard deviation.

**Table 2:** Characteristics of the study participants according to sex.

Variables		*n* (%)		*t*-Value	*p*-Value
	Male (*n* = 152)	Female (*n* = 204)	Total (*n* = 356)		
Height (m)					
Mean ± SD	1.64 ± 0.78	1.55 ± 0.06	1.59 ± 0.08	−10.241	<0.001
Weight (kg)					
Mean ± SD	55.83 ± 12.75	51.71 ± 12.93	53.47 ± 12.99	−2.991	0.003
Waist circumference (cm)					
Mean ± SD	72.40 ± 9.86	68.85 ±10.72	70.37 ± 10.50	−3.198	0.002
BMI-for-age (*z*-score)					
Mean ± SD	0.15 ± 1.38	0.08 ± 1.38	0.11 ± 1.38	−0.488	0.626
Severe thinness	0 (0.0)	1 (0.5)	1 (0.3)		
Thinness	7 (4.6)	9 (4.4)	16 (4.5)		
Normal	99 (65.1)	141 (69.1)	240 (67.4)		
Overweight	30 (19.7)	30 (14.7)	60 (16.9)		
Obese	16 (10.5)	19 (9.3)	35 (9.8)		
Severely obese	0 (0.0)	4 (2.0)	4 (1.1)		
Abdominal obesity					
At risk	21 (13.8)	31 (15.2)	52 (14.6)		
Not at risk	131 (86.2)	173 (84.8)	304 (85.4)		
Binge eating behavior					
Mean ± SD	9.51 ± 6.53	10.93 ± 6.06	10.32 ± 6.29	2.116	0.035
No	133 (87.5)	173 (84.8)	306 (86.0)		
Moderate	17 (11.2)	28 (13.7)	45 (12.6)		
Severe	2 (1.3)	3 (1.5)	5 (1.4)		

SD = standard deviation.

**Table 3:** Factors associated with binge eating behavior among adolescents.

Variables	Simple Linear Regression			Multiple Linear Regression		
	*β*	*t*	*p*	*β*	*t*	*p*
**Demographic and socioeconomic factors**						
Age	0.020	0.368	0.713	-	-	-
Parental income	−0.077	−1.447	0.149	-	-	-
Household size	0.005	0.095	0.924	-	-	-
**Family factors**						
Priority of family meals	−0.083	−1.575	0.116	-	-	-
Atmosphere at family meals	−0.044	−0.827	0.409	-	-	-
Structure at family meals	0.061	1.142	0.254	-	-	-
Family cohesion	−0.244	−4.742	<0.001	−0.206	−4.190	<0.001
**Psychological factors**						
Perception of body size	0.186	3.553	<0.001	0.157	3.183	0.002
Depressive symptoms	0.263	5.126	<0.001	0.194	3.785	<0.001
Self-esteem	−0.243	−4.713	<0.001	−0.154	−3.017	0.003
Perfectionistic self-presentation	0.065	1.232	0.219	-	-	-
**Body weight status**						
BMI-for-age	0.127	2.415	0.016	-	-	-
Waist circumference	0.154	2.927	0.004	-	-	-

The variables that produced *p* < 0.25 in the simple linear regression model were chosen to be included in the multiple linear regression analysis. Multiple linear regression model: *R*^2^ = 0.177, Adjusted *R*^2^ = 0.165, *F* = 15.056, *p* < 0.001. Furthermore, all the results were adjusted to take into account sex of the participants.
